# 805. Outbreak of *Ralstonia pickettii* Bacteremia Caused by Contaminated Hydromorphone in a Universitary Hospital in Bogota, Colombia

**DOI:** 10.1093/ofid/ofab466.1001

**Published:** 2021-12-04

**Authors:** Shirley Vanessa Correa Forero, Ivonne Tatiana Ordoñez Blanco, Samuel Martinez-Vernaza, Sandra Liliana Valderrama-Beltrán, Gloria Cortes, Claudia Janneth Linares Miranda, Angela Patricia Gonzalez Rubio, Viviana Andrea Pinzon Garcia

**Affiliations:** 1 Hospital Universitario San Ignacio, Bogota, Distrito Capital de Bogota, Colombia; 2 Hospital Universitario San Ignacio - Pontificia Universidad Javeriana, Bogota, Distrito Capital de Bogota, Colombia

## Abstract

**Background:**

***Ralstonia pickettii*** are aerobic non fermenter gram negative bacilli isolated in water and soil. It is related to nosocomial infection outbreaks and considered an opportunistic pathogen. There have been outbreaks reports due to contaminated water systems and sterile drug solutions which mainly occurs during manufacturing. We present the report of an outbreak of *R. pickettii* bacteremia secondary to a contamination of hydromorphone vials.

**Methods:**

In February 2021 an outbreak of *R. pickettii* bacteremia was identified. All isolates were from blood cultures with slow growth, thus indicating the culturing of liquid inputs, intravenous administration solutions and commonly used drugs among patients including hydromorphone. Mass spectrometry (MALDI-TOF) was used for the identification and automated microdilution to determine sensitivity to antimicrobials of the isolates and clonality analysis of genetic relationships was carried out using the DICE coefficient, UPGMA algorithm

**Results:**

During the outbreak, 19 patients with *R. pickettii* bacteremia were identified The global attack rate was 1,9%. 11/19 (58%) were women and 13/19 (68%) of the isolations were from inward patients and 6/19 (32%) were from intensive care unit. Factors that could contribute to the appearance of the outbreak were underlying pathology, 2 patients with a diagnosis of diabetes mellitus, 10 patients with a diagnosis of arterial hypertension, 5 patients with obesity, 6 patients with heart disease, additionally 7 patients with a diagnosis of SARS COV 2 and 6 patients with the use of corticosteroids. The global attack rate was 1,9% and mortality was 31.5% (6 patients). R. pickettii was identified from two batches of hydromorphone by MALDI-TOF and the clonality study concluded that the isolates analyzed, were clonal with a 100% similarity. The associated mortality rate was 5/29 (26.3%).

**Conclusion:**

We confirmed an outbreak of R. pickettii due to the contamination of two hydromorphone badges in Colombia. It is crucial to acknowledge the importance of infection control and surveillance during the COVID-19 pandemic as well as maintaining adequate quality control of medication production in order to avoid presenting this kind of outbreaks.

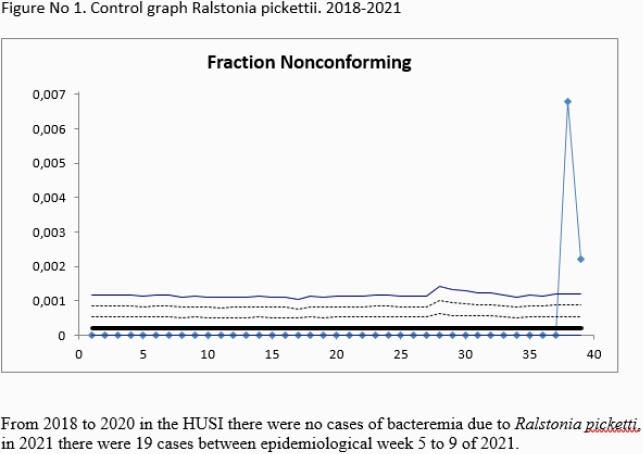

**Disclosures:**

**Sandra Liliana. Valderrama-Beltrán, MD, MSc**, **Biotoscana** (Speaker’s Bureau)**MSD** (Grant/Research Support, Scientific Research Study Investigator, Research Grant or Support, Speaker’s Bureau)**Pfiezer** (Speaker’s Bureau)

